# EMBL’s European Bioinformatics Institute (EMBL-EBI) in 2023

**DOI:** 10.1093/nar/gkad1088

**Published:** 2023-11-28

**Authors:** Matthew Thakur, Annalisa Buniello, Catherine Brooksbank, Kim T Gurwitz, Matthew Hall, Matthew Hartley, David G Hulcoop, Andrew R Leach, Diana Marques, Maria Martin, Aziz Mithani, Ellen M McDonagh, Euphemia Mutasa-Gottgens, David Ochoa, Yasset Perez-Riverol, James Stephenson, Mihaly Varadi, Sameer Velankar, Juan Antonio Vizcaino, Rick Witham, Johanna McEntyre

**Affiliations:** Data Services Teams, EMBL’s European Bioinformatics Institute (EMBL-EBI), Wellcome Genome Campus, Hinxton CB10 1SD, UK; Open Targets, EMBL’s European Bioinformatics Institute (EMBL-EBI), Wellcome Genome Campus, Hinxton CB10 1SD, UK; Training Team, EMBL’s European Bioinformatics Institute (EMBL-EBI), Wellcome Genome Campus, Hinxton CB10 1SD, UK; Training Team, EMBL’s European Bioinformatics Institute (EMBL-EBI), Wellcome Genome Campus, Hinxton CB10 1SD, UK; Industry Partnerships, EMBL’s European Bioinformatics Institute (EMBL-EBI), Wellcome Genome Campus, Hinxton CB10 1SD, UK; Data Services Teams, EMBL’s European Bioinformatics Institute (EMBL-EBI), Wellcome Genome Campus, Hinxton CB10 1SD, UK; Open Targets, EMBL’s European Bioinformatics Institute (EMBL-EBI), Wellcome Genome Campus, Hinxton CB10 1SD, UK; Data Services Teams, EMBL’s European Bioinformatics Institute (EMBL-EBI), Wellcome Genome Campus, Hinxton CB10 1SD, UK; Industry Partnerships, EMBL’s European Bioinformatics Institute (EMBL-EBI), Wellcome Genome Campus, Hinxton CB10 1SD, UK; Data Services Teams, EMBL’s European Bioinformatics Institute (EMBL-EBI), Wellcome Genome Campus, Hinxton CB10 1SD, UK; Data Services Teams, EMBL’s European Bioinformatics Institute (EMBL-EBI), Wellcome Genome Campus, Hinxton CB10 1SD, UK; Training Team, EMBL’s European Bioinformatics Institute (EMBL-EBI), Wellcome Genome Campus, Hinxton CB10 1SD, UK; Open Targets, EMBL’s European Bioinformatics Institute (EMBL-EBI), Wellcome Genome Campus, Hinxton CB10 1SD, UK; Industry Partnerships, EMBL’s European Bioinformatics Institute (EMBL-EBI), Wellcome Genome Campus, Hinxton CB10 1SD, UK; Open Targets, EMBL’s European Bioinformatics Institute (EMBL-EBI), Wellcome Genome Campus, Hinxton CB10 1SD, UK; Data Services Teams, EMBL’s European Bioinformatics Institute (EMBL-EBI), Wellcome Genome Campus, Hinxton CB10 1SD, UK; Data Services Teams, EMBL’s European Bioinformatics Institute (EMBL-EBI), Wellcome Genome Campus, Hinxton CB10 1SD, UK; Data Services Teams, EMBL’s European Bioinformatics Institute (EMBL-EBI), Wellcome Genome Campus, Hinxton CB10 1SD, UK; Data Services Teams, EMBL’s European Bioinformatics Institute (EMBL-EBI), Wellcome Genome Campus, Hinxton CB10 1SD, UK; Data Services Teams, EMBL’s European Bioinformatics Institute (EMBL-EBI), Wellcome Genome Campus, Hinxton CB10 1SD, UK; Data Services Teams, EMBL’s European Bioinformatics Institute (EMBL-EBI), Wellcome Genome Campus, Hinxton CB10 1SD, UK; Data Services Teams, EMBL’s European Bioinformatics Institute (EMBL-EBI), Wellcome Genome Campus, Hinxton CB10 1SD, UK

## Abstract

The European Molecular Biology Laboratory's European Bioinformatics Institute (EMBL-EBI) is one of the world's leading sources of public biomolecular data. Based at the Wellcome Genome Campus in Hinxton, UK, EMBL-EBI is one of six sites of the European Molecular Biology Laboratory (EMBL), Europe's only intergovernmental life sciences organisation. This overview summarises the latest developments in the services provided by EMBL-EBI data resources to scientific communities globally. These developments aim to ensure EMBL-EBI resources meet the current and future needs of these scientific communities, accelerating the impact of open biological data for all.

## Graphical Abstract

**Graphical Abstract:**
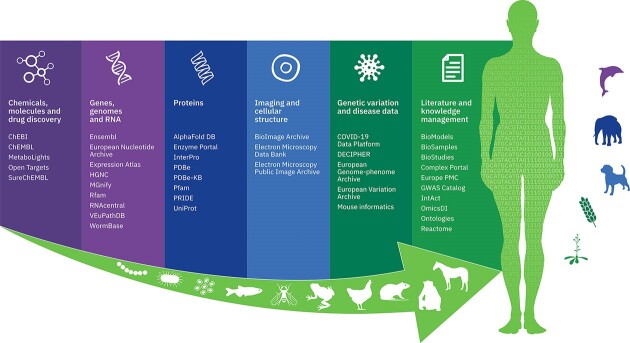

## Introduction

The European Molecular Biology Laboratory's European Bioinformatics Institute (EMBL-EBI) is one of the world's leading sources of public biomolecular data. Based at the Wellcome Genome Campus in Hinxton, UK, EMBL-EBI is one of six sites of the European Molecular Biology Laboratory’ (EMBL), Europe's only intergovernmental life sciences organisation, whose research infrastructure and services support cutting-edge science globally. EMBL-EBI is contributing to EMBL’s 2022–2026 ‘Molecules to Ecosystems’ programme, which aims to establish the molecular basis of life in context, to gain new knowledge that is relevant to understanding life on Earth, and to provide translational potential to support advances in human and planetary health.

EMBL-EBI enables life science research and its translation to medicine, agriculture, industry and society by:

- freely providing data and bioinformatics services to the scientific community in ways that promote scientific progress,
- contributing to the advancement of biology through investigator-driven research,
- providing bioinformatics training to scientists at all levels,
- disseminating cutting-edge technologies to industry and applications of science,
- supporting, as an ELIXIR Node, the coordination of biomolecular data provision in Europe.

This overview focuses on services that EMBL-EBI data resources provide to scientific communities globally, describing related training and industry applications where relevant. Many other EMBL-EBI data resources have dedicated articles elsewhere in this special issue, so this overview focuses on major changes to data resources not described elsewhere.

EMBL-EBI data resources comprise: deposition databases, which archive experimental data; added-value databases, which provide annotation, curation, reanalysis and integration of deposited data; and open source software tools, that enable reuse of these resources. Deposition databases, added-value databases and tools are described and accessed via the EMBL-EBI services web portal. All EMBL-EBI data resources and many software systems can be downloaded and installed locally, and are made available on an open and free basis for reuse. Many services offer further bulk and machine-readable access including via API, FTP, Aspera and Globus services.

EMBL-EBI resources serve as foundations for hundreds of external resources, research programmes and tools, with many recent developments described below. In December 2022, the Global Biodata Coalition, a forum for research funders which aims to coordinate worldwide biodata infrastructure and ensure its sustainable financial support, identified their Global Core Biodata Resources (GCBRs) – a collection of data resources recognised as critical to life science and biomedical research worldwide. 16 of the 37 GCBRs identified in 2022 are hosted at EMBL-EBI or involve the organisation as a partner.

An emerging theme in many of the updates described below is the increasing need for integrated visualisation and analysis across multiple sources and modalities of data in order to examine the most cutting edge research questions. The UniVar resource described below shows the impact integration can have, bringing together genetic, macromolecular structure and protein annotation to offer users the most comprehensive understanding of the impact of variation on function. The PRIDE (PRoteomics IDEntifications) database also describes innovations in integrated proteogenomics functionality below.

The proliferation of open data practice and global data resources, while providing greater choice for users, also creates challenges in reaching the findability, accessibility, interoperability and reusability required to create impact. The 3D Beacons Network reported below is one approach to make disparate sources of data easier for users to find and access. Work on adopting standard mass spectrometry Universal Spectrum Identifiers by PRIDE and the large biological imaging datasets OME-Zarr standard by BioImage Archive, lay the groundwork required for interoperability and reuse. Well-organised, high-quality, curated data resources will continue to be of crucial importance for developers of AI applications, demonstrated by the computer vision and machine learning-friendly dataset discover features reported below by BioImage Archive, and summarised at institutional level on the EMBL-EBI AI Hub website.

### The Impact of EMBL-EBI data resources

EMBL-EBI tracks the use of data resources through metrics including the number of web requests and unique IP addresses visiting service websites, the volume of data deposited, and the number of open citations EMBL-EBI data resources receive in scientific publications. While each metric has limitations and cannot provide an exact quantification of use, considered together they give an indication of the scale and trend in usage.

**Figure 1.**
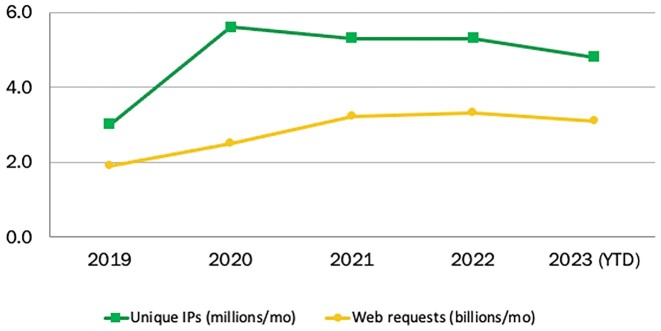
Unique IP visits (green) and web requests (yellow) to EMBL-EBI data resources, 2019–2023.

EMBL-EBI data resources have a global reach, with every UN member state country represented in our user base. Recent trends in usage show that demand from researchers for EMBL-EBI data resources, which spiked during early stages of the COVID-19 pandemic in 2020, remained much higher in 2023 than 2019 pre-pandemic levels, with just under 5 million unique users every month (Figure 1). Regarding how researchers access resources, in recent years researchers are making greater use of bulk- and machine-readable access methods.

**Figure 2.**
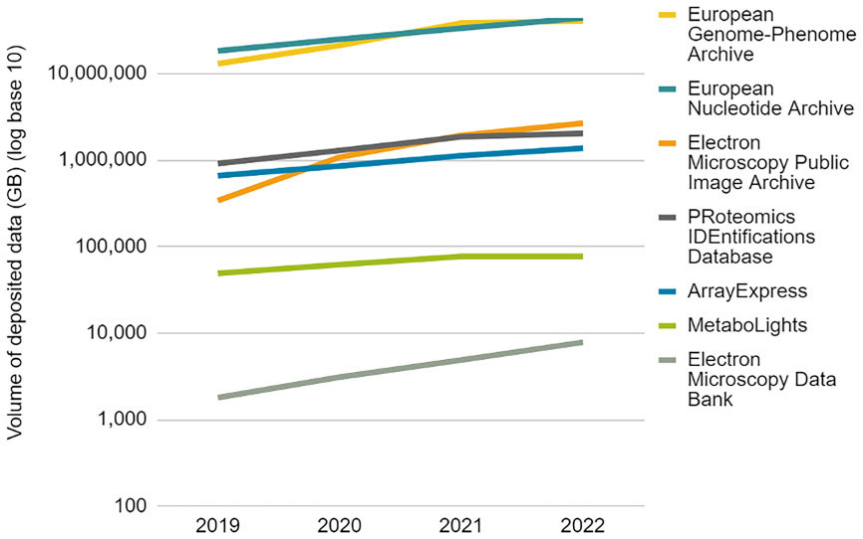
Annual deposition into seven deposition database resources. Note the log scale and rapid rate of growth for electron microscopy resources in this period.

The rate of data deposition by volume into EMBL-EBI’s archival resources continues to accelerate, with active storage now over 90 Petabytes (Figure 2). The two largest archival resources are European Nucleotide Archive (ENA) (1) and European Genome-phenomeArchive (EGA) (2), between them accounting for over 90% of total data deposited to date. Notably rapid data growth in recent years has been in imaging data resources, including the electron microscopy imaging resources Electron Microscopy Public Image Archive (EMPIAR) (3) and Electron Microscopy Databank (EMDB) (Turner and wwPDB Consortium, 2023, PPR: PPR738258).

## Major changes in the EMBL-EBI data resource portfolio

### ProtVar integrates amino acid information to facilitate interpretation of missense variants

**Figure 3.**
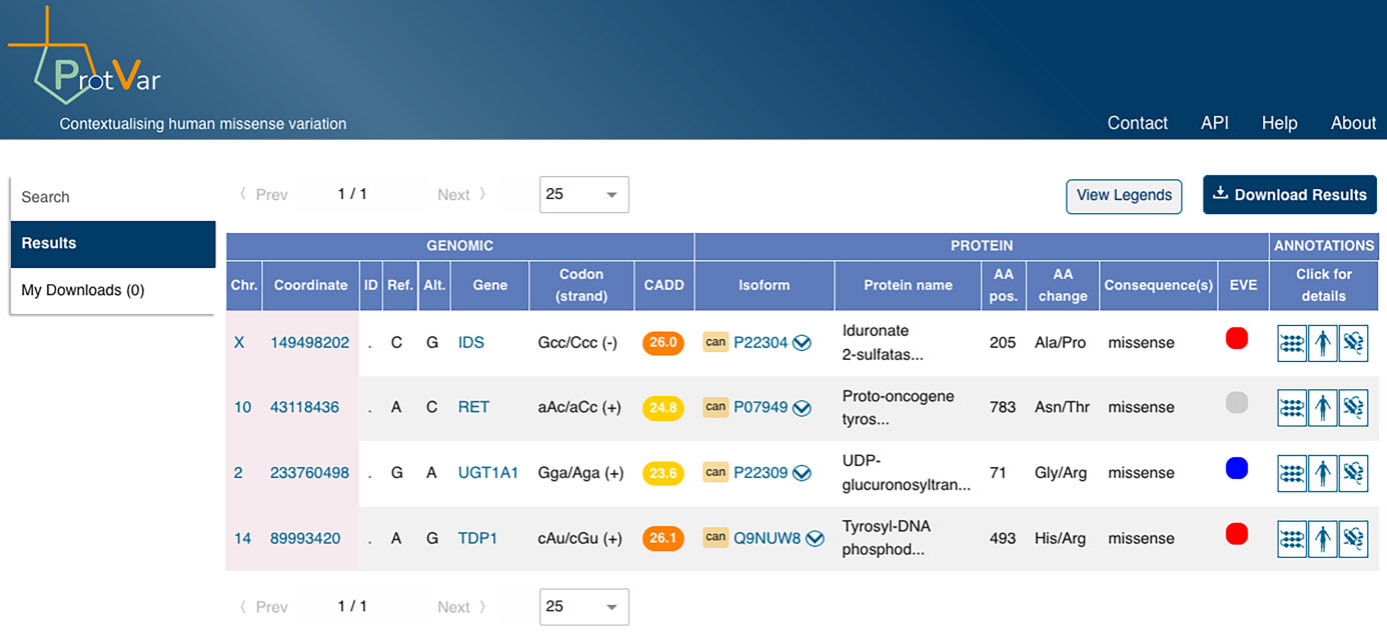
Example search results in the PRotVar user interface for submitting variants in genomic or protein formats. Also available via API.

Missense variation is a point mutation in which a single nucleotide change results in a different amino acid within a protein, leading to complex downstream effects on phenotype, disease susceptibility and drug response. In May EMBL-EBI launched the ProtVar tool, a new service for mapping and annotating variants at a per-residue level to contextualise and evaluate human missense variation in proteins (Figure 3). Protein functional annotations, both curated and automatically generated, are imported from UniProt (4). Protein structures and structural annotations are from Protein DataBank Europe (PDBe) (5) and AlphaFold (6). Variants and relevant regions are shown on protein 3D models using an interactive viewer from PDBeMol*. Pathogenicity predictors are from the Evolutionary model of Variant Effect (EVE) (7) and Combined Annotation Dependent Depletion (CADD) (8) tools and conservation is from VarSite (9). Co-located variants at the residue level are retrieved from GnomAD (Chen et al., 2022, PPR: PPR556342), ClinVar (10), COSMIC (11) and other sources mapped to proteins by UniProt. Predictions based on structures such as predicted pockets, stability and interfaces come from a collaboration with Open Targets (12). The results are browsable in the ProtVar user interface, downloadable and available programmatically via an API. It allows research, industry and clinical genetics users to rapidly identify the potential effects of missense variation on human health and disease, with potential applications in drug discovery.

### Making experimentally determined and computationally predicted molecular structures FAIRer through the 3D-Beacons Network

**Figure 4.**
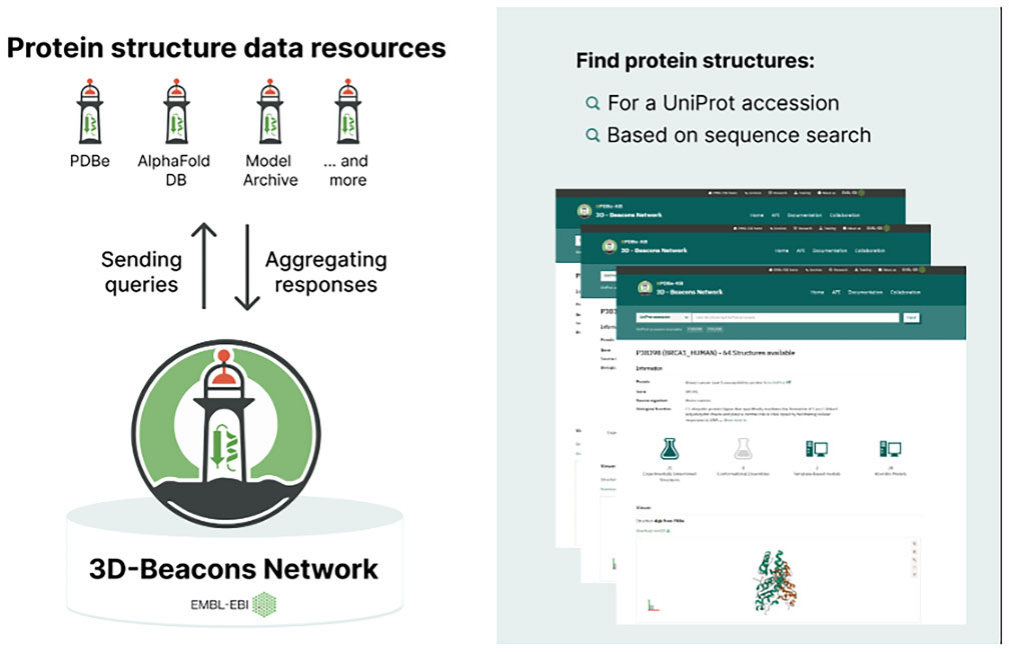
Overview of the 3D-Beacons Network, a new data sharing platform of EMBL-EBI.

In 2021, EMBL-EBI furthered its commitment to data democratisation by establishing the AlphaFoldProtein Structure Database (6) in collaboration with Google DeepMind, which aimed to lower barriers to accessing protein structure models predicted by cutting-edge tools like AlphaFold 2. Building upon these efforts, the PDBe team at EMBL-EBI launched the 3D-Beacons Network (13) in 2022, a platform that serves as an open collaboration between providers of macromolecular structure models (Figure 4). This network aims to offer model coordinates and meta-information from all contributing data resources in a standardised format, making it a unified platform for diverse structural data. Significantly, the 3D-Beacons Network improves the FAIRness (Findability, Accessibility, Interoperability and Reusability) of both experimentally determined and computationally predicted protein structures. It is a one-stop solution for accessing structures of monomeric proteins and molecular assemblies from various providers, including PDBe, the Protein Ensemble Database (14), the Small-Angle Scattering Biological Data Bank (15), AlphaFold DB, Swiss-Model (16), Model Archive (17) and others. The network is centred around a hub API that users interact with. The hub sends queries to data providers, aggregates their responses and presents the data to users in standardised format. The establishment of the 3D-Beacons Network underscores the pivotal role EMBL-EBI continues to play in providing FAIR access to life-science data at scale.

### Enabling Web and Programmatic Access through a renewed Job Dispatcher

The Job Dispatcher tools framework (18) provides an easy way for users to access EMBL-EBI’s most popular bioinformatics sequence analysis applications and sequence libraries at scale, offering public access to EMBL-EBI’s high-performance computing clusters. User-friendly web interfaces and established RESTful and SOAP Web Services Application Programming Interfaces (APIs) allow integration into third-party systems. Job Dispatcher powers various popular sequence analysis services hosted at the EBI, including InterProScan, UniProt and Ensembl Genomes (19).

2023 saw the launch of a new Job Dispatcher beta site. The new website reorganises and revamps the tool and documentation pages as well as adding new features. A new text field allows users to search for job results and gives improved information on job status. A blog section provides a channel for EMBL-EBI to share the latest news and data/tool updates. Extensive help documentation on the web and programmatic access is provided, as well as entry points to our related training and outreach activities.

## New features and applications of existing data resources

### PRIDE promotes growth in proteomics data and integration into other resources

The PRIDE (PRoteomicsIDEntifications) database (20) is the world-leading resource for mass spectrometry (MS)-based proteomics datasets and continues to lead the International ProteomeXchange Consortium (21), which is one of the Global Core Biodata Resources. On average ∼515 datasets per month were submitted to PRIDE so far in 2023. This has been achieved thanks to increased automation of data submissions, dramatically decreasing the average time required to get an accession number from 34 h to 4 min. Additionally, other improvements in the PRIDE infrastructure have provided improved functionality for accessing mass spectra data using the standard Universal Spectrum Identifiers (22).

Furthermore, the PRIDE team is increasingly reusing and reanalysing public proteomics datasets and integrating the results in other EMBL-EBI resources, so that proteomics data is made more accessible to biologists and clinicians. Some recent examples of data reuse include: (i) the integration of quantitative proteomics datasets in Expression Atlas for human, mouse and rat baseline tissue data (23,24); (ii) the ‘PTMeXchange’ project, for making post-translational modifications (PTM) more FAIR, by linking the information between PRIDE and UniProt and (iii) linking proteomics and genomics/transcriptomics information by using proteogenomics approaches in different contexts, involving resources such as Ensembl (25) and MGNify (26).

### Interactive galleries and AI-ready datasets at BioImage Archive

**Figure 5.**
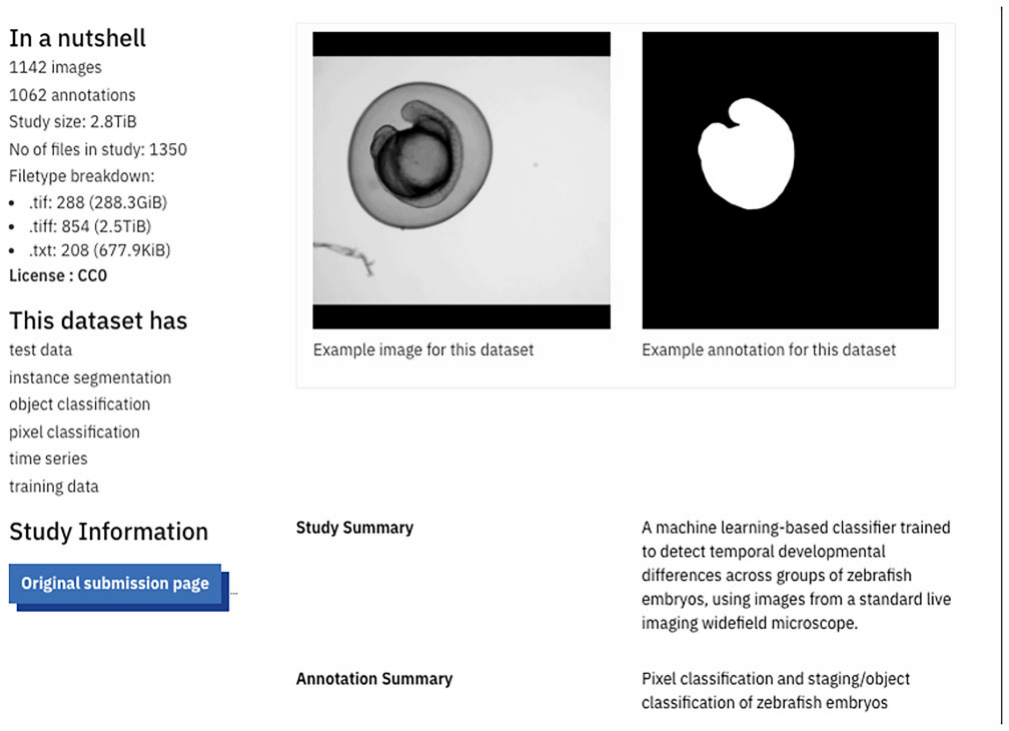
A Zebrafish imaging dataset visualised in BioImage Archive's new AI-ready gallery function. In line annotations show the staging/object classification required to train supervised AI models.

The BioImage Archive (27), dedicated to storing and distributing biological images, recently launched two interactive galleries of images from its collection, one showing a range of both visually and biologically interesting images chosen for diversity of imaging techniques and biological relevance, and a second gallery of ‘AI-ready’ image datasets (Figure 5). This latter collection presents both images and the whole-image annotation files that are required to train supervised AI models. Images are made available in a consistent format, OME-Zarr (28), the emerging standard for sharing large biological imaging datasets. Both images and annotations are accompanied by detailed metadata, following REMBI guidelines (29) for images and new standards for image annotations developed at a community workshop organised by EMBL-EBI in January 2023. The data presentation format is specifically tailored to meet the needs of the machine learning and computer vision communities. These collections will grow over time, as datasets suitable for reuse for AI models are deposited in the Archive.

### More powerful integrated target-disease association evidence via Open Targets

The Open Targets consortium brings together academic (EMBL-EBI and the Wellcome Sanger Institute) and pharmaceutical partners (GSK, Pfizer, Genentech, BMS, Sanofi) with the aim of systematically identifying and prioritising potential therapeutic drug targets. The Open Targets informatics ecosystem provides open source tools and resources that support this goal for the wider scientific community (30).

**Figure 6.**
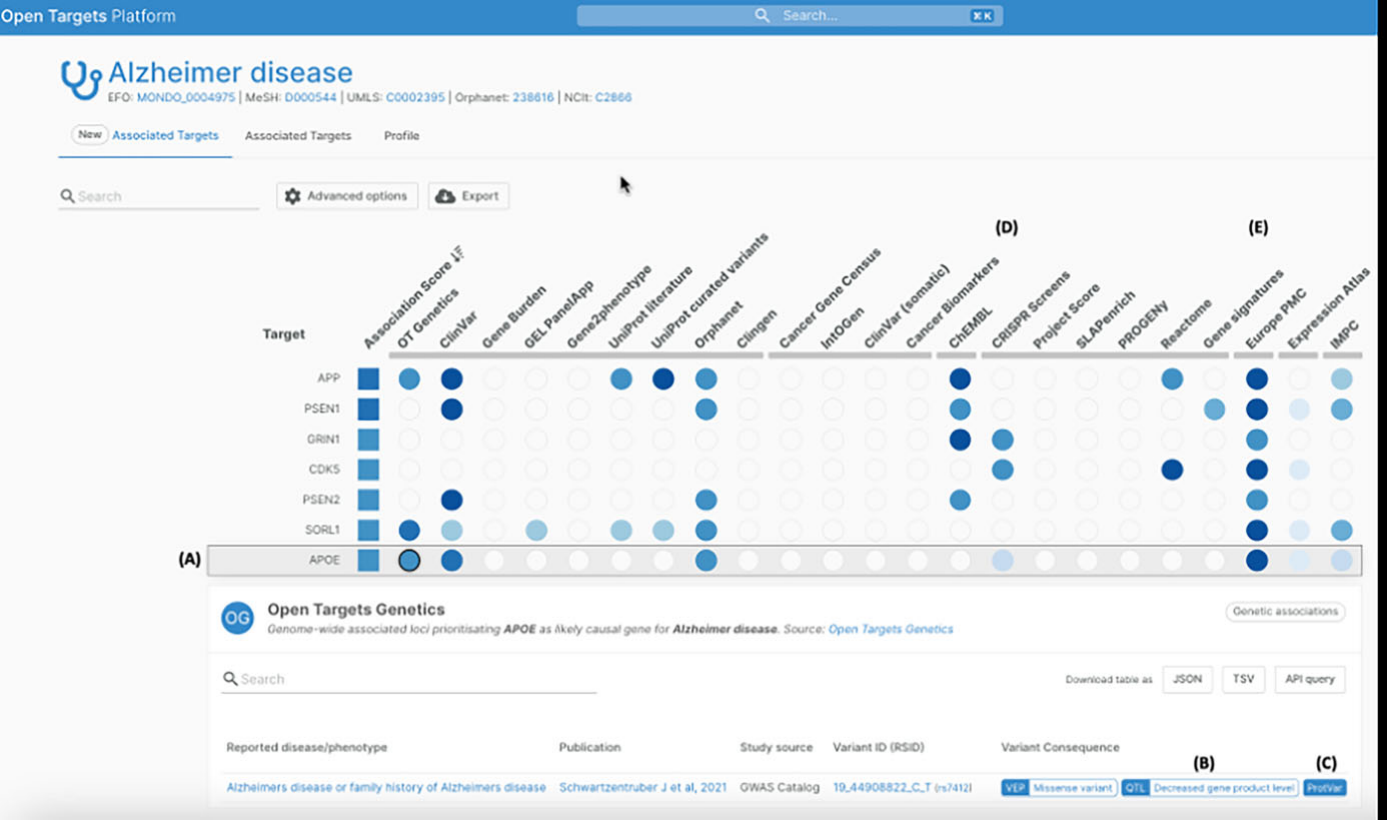
The Open Targets Platform newly redesigned target-disease associations page. Example page shown is diseases and traits associated with the target PCSK9. Annotations A-E designate features of interest described in the text (https://platform.opentargets.org/target/ENSG00000169174/associations).

In 2023 the Open Targets Platform (https://platform.opentargets.org/) launched a new interface design that enables users to prioritise target-disease evidence data sources dynamically, and rapidly access the supporting evidence for any target-disease association (Figure 6A). New features allow users to better understand the effect of genetic variants and consequence of gene perturbation, via integration of protein function context from the new EBI ProtVar resource (above and 6B, https://www.ebi.ac.uk/ProtVar/), Quantitative Trait Loci (QTL)-based direction of effect from Open Targets Genetics (Figure 6C, https://genetics.opentargets.org/), new data from CRISPRbrain screens (Figure 6D) (31) and Cancer DepMap gene essentiality screens (32).

Extraction of target–disease evidence from the scientific literature was enhanced by extending text-mining to preprints and patents in collaboration with EMBL-EBI’s Europe PMC (Figure 6E). A new feature utilises OpenAI’s GPT-3 to provide contextual summaries of the relationship between targets and diseases from the literature evidence. User interface search functionality redesign has improved data findability. A manifest file with richer metadata provenance is now made available to users with every Platform release, enhancing our commitment to FAIR principles. Backend improvements to our literature knowledge extraction pipeline have boosted performance. Furthermore, we have strengthened our commitment to support open access research by marking the Open Targets Platform with a CC0 licence, allowing downstream users to consume the data without restriction.

## Industry

EMBl-EBI’s Industry Programme engages multi-national research-intensive companies who make significant use of EMBL-EBI data and resources. It operates via a subscription-based model and has now grown, in its 27th year, to include 30 companies, representing most of the top 20 pharmaceutical companies as well as major agri-food, nutrition, and healthcare companies. The programme is science-based and centred on principles of precompetitive collaboration and discussion.

For EMBL-EBI, the programme provides regular contact and interaction with key stakeholders and opinion leaders at major life sciences companies, which in turn helps to improve the utility of EMBL-EBI data and services. One significant element of the programme are bespoke knowledge exchange workshops focussed on topics proposed by the membership.

The Industry Programme delivers 10–12 workshops each year,in the US, Japan and UK. The workshops bring cutting edge research from leading academics to front-line industry scientists and provide a neutral pre-competitive environment for industry-based scientists to share peer-to-peer learning, which frequently surfaces common challenges and opportunities to address them collectively. Driven by a shared interest to optimise drug discovery and development, recent workshop themes have included the application of artificial intelligence / machine learning, use of biobanks, latest developments in genomic technologies, applications of AlphaFold, digital biomarkers in clinical research, knowledge management systems and the use of single-cell genomics.

Where relevant, review articles and whitepapers/position statements have been published as direct outputs from relevant workshops. These include industry analysis of drug-discovery informatics applications of specific tools such as single-cell sequencing (33), machine learning (34), text mining (35), Patient-Derived Tumor Xenograft Models (36) and antibody informatics (37). In other cases industry partners have collaborated on new ontologies and standards of widespread utility, including in toxicology (38,39), and Bioactive entity metadata (40).

Partners from the agri-food sector (Bayer CropScience, Syngenta and Unilever), are currently working with EMBL-EBI on plans to design and build an AgriData Platform. To achieve this, in 2023 we launched a dedicated agricultural technology consortium including academics and companies. The proposed EMBL-EBI AgriData platform will be a unified public data platform for agricultural genomes and environments (e.g. crops, livestock, associated pests and pathogens, field soils and water), incorporating computational tools for data analysis. Based on experience with the OpenTargets platform, we are confident that the AgriData platform will be an asset that aggregates and integrates existing public data while fostering much needed common standards for data sharing.

In future, the EMBL-EBI Industry Programme will continue to evolve according to the needs of its members, always with a vibrant schedule of knowledge exchange workshops at its core. Involvement will increasingly extend beyond EMBL-EBI to include all EMBL sites, and there will always be provision to accommodate a low carbon footprint by delivering all workshops in hybrid format.

## Training

EMBL-EBI’s training programme empowers scientists to get the most out of openly accessible data resources and services, and to develop key bioinformatics analysis skills. Fundamental principles for FAIR and open data management are incorporated into all live courses. Community engagement is key to the ongoing evolution of our portfolio, which incorporates a significant contribution to EMBL’s Course and Conference programme and a dynamic, community-driven offering through externally funded collaborations. Roughly 500 scientists participate in in-person courses per year, with the majority of these reporting that they go on to pass on their learning to others. Web-based on-demand training sees around 500000 unique IP users per year. We also offer support for trainers – within EMBL and externally through our grant-funded projects and other collaborations.

On-demand training includes a growing catalogue of curated collections and learning pathways that provide trainees with a structured approach to learning on a given topic. An openly accessible training material set is available for each course, radically improving the FAIRness (especially the findability) of live course materials. Those undertaking self-directed learning through EMBL-EBI on-demand materials can track their progress, keep a record of courses they have completed (including live courses – certificates of course completion are now issued through their ‘myLearning’ account), and plan their future studies. MyLearning account holders can now create and share playlists of courses. This feature was introduced in 2023 with higher education in mind, enabling teaching staff to create a bespoke collection of practical courses for their students and share them effortlessly.

EMBL-EBI hosts the Competency Hub—a repository of frameworks that define the knowledge, skills and attitudes required by professionals in a specific field and relate them to relevant training resources and career profiles. In 2023, we routinely began to provide links from EMBL-EBI courses to relevant competency frameworks; users can either start with a competency listed in the competency hub and use it to link through to any EMBL-EBI courses that contribute towards the development of that competency, or access information on the ‘Competencies’ tab of any EMBL-EBI course to find a list of competencies developed.

EMBL-EBI’s courses are taught by an extensive faculty, comprising both EMBL staff and guest faculty members. To recognise the contributions of our faculty, we have developed a ‘Meet our Trainers’ page, introducing each faculty member and pointing to the course or courses that they contribute to.

New content developed and made openly available in 2023 has included courses developed as part of several community-led projects. For example, Practical Biocuration was developed through the ELIXIR-CONVERGE project, a self-paced online tutorial on federated data analysis, was created as part of the CINECA project, and a new scholarship programme to develop computational haematologists has been developed in collaboration with the European Hematology Association. In addition to serving the needs of a varied and ever-diversifying user community, the training programme draws inspiration from EMBL’s scientific Programme, Molecules to Ecosystems. Webinar series highlighting EMBL’s new transversal themes include a new series on the Molecular Building Blocks of Life and a recently completed series on Plants: a data sciences perspective.

## Conclusion

At the heart of EMBL-EBI data resources are the submission databases that host and share experimentally generated data from the molecular biology community. The resources and developments described here and elsewhere in this issue are built on these foundations. The critical importance of many of these resources in underpinning research internationally has recently been recognised by the Global Biodata Coalition. The goal, however, is to make sure these individual entities are able to provide a comprehensive and integrated view of molecular biology across scales and modalities, in collaboration with global partners, to support research and data science that impacts on global challenges.

## Data Availability

All of the data resources described above are freely available to access and reuse at https://www.ebi.ac.uk/services.
